# H-type Rectovestibular Fistula Presenting as a Left Labial Abscess: A Diagnostic Masquerade

**DOI:** 10.7759/cureus.79622

**Published:** 2025-02-25

**Authors:** Adam Amir

**Affiliations:** 1 General Surgery, Malacca General Hospital, Malacca, MYS

**Keywords:** anorectal malformations, ectopic anal opening, h-type fistula, labial abscess, rectovaginal fistula

## Abstract

Anorectal malformations (ARMs) is a congenital abnormality that is commonly seen in newborns without a normal or ectopic anal opening. H-type fistula is a rare subtype and is commonly diagnosed in females after a history of passing stool from the vagina which may be the first and only sign of an underlying ARM which necessitates further diagnostic modalities, treatment, and follow-up. These children may have a normal anal opening. We report on a rare case of rectovaginal fistula presenting with a left labial abscess.

## Introduction

Anorectal malformations (ARMs) affect approximately 1 in every 2000 to 5000 live births [1,2]. This often refers to a condition where there is an imperforate or ectopic anal opening. However, there is a wide spectrum of presentations from low anomalies with perineal fistula to high complicated anomalies. Its aetiology is still not fully known and is likely to be multifactorial [2]. Diagnosis is often made at birth where there is an absent anal opening; however, certain types of malformations may go undiagnosed till infancy or early childhood. H-type fistula is a rare subtype of ARM with an external anal opening in the normal or ectopic position. Its occurrence is reported to be 0.1-16% of all ARMs [3]. It occurs more commonly in females and diagnosis is often missed at birth, often only being recognised when faeces is observed passing through the vagina.

## Case presentation

The patient was a one-month-old baby girl born at full term via vaginal delivery. Antenatal history was unremarkable. She had a single admission to the paediatrics unit before for neonatal jaundice requiring phototherapy. There were no concerns about any gross anatomical abnormalities. The patient’s mother reported that the patient was otherwise well until three days prior to the presentation. The child had started to develop a progressive swelling over the left labia which became erythematous. She was otherwise well and not septic.

**Figure 1:**
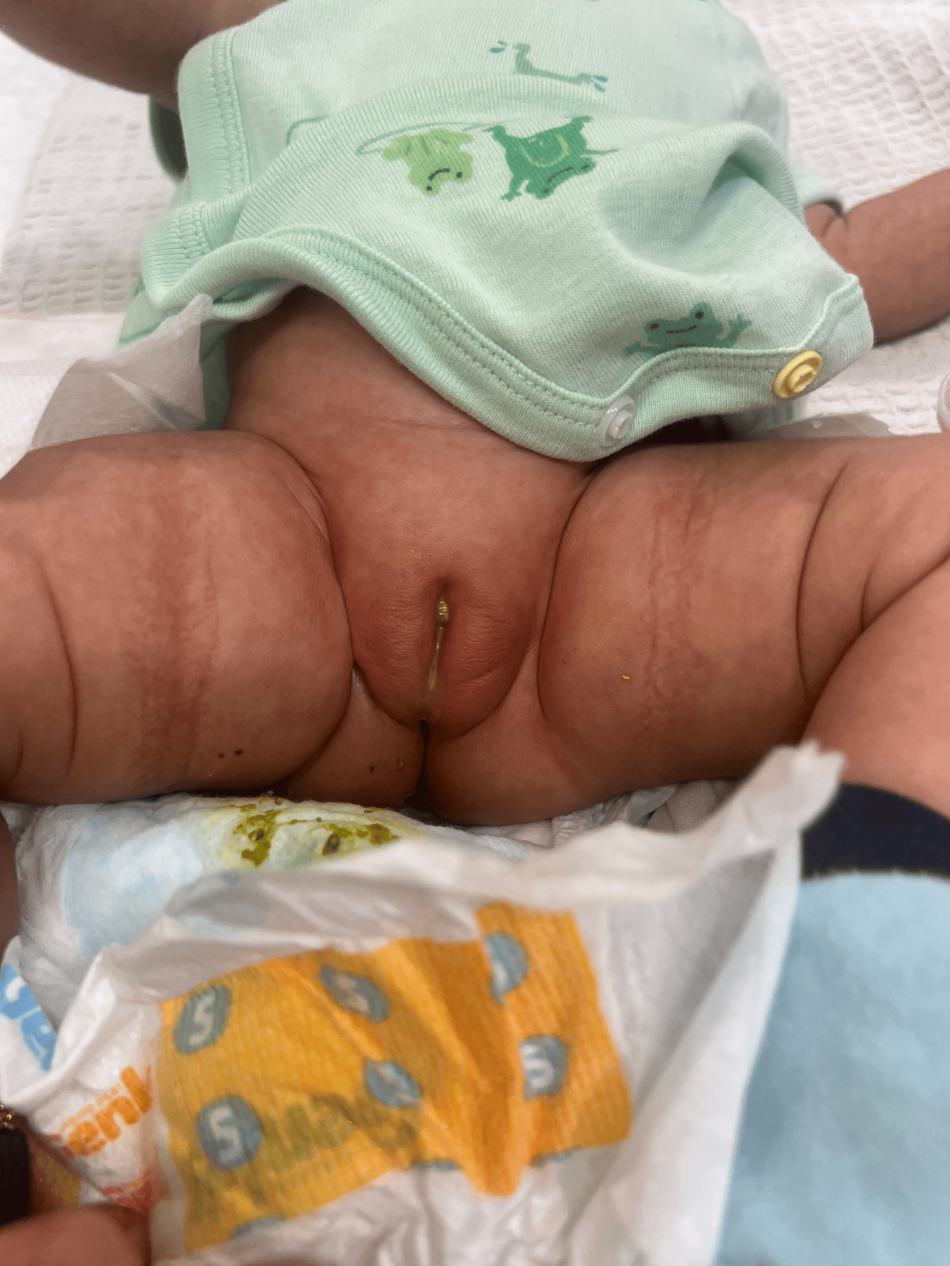
Presence of faecal matter within the vestibule; the left labia appears oedematous and erythematous.

After three days of unresolved symptoms, the mother brought the patient to our centre to seek treatment. Our team was initially consulted for a left labial abscess. On examination, the child did not appear septic and was not dysmorphic. The left labia appeared oedematous and erythematous. It was fluctuant without any obvious punctum. On separating the labia, it was noted that there was faecal matter within the vaginal opening (Figure 1). No visible sinus was seen. Prior to the presentation, the patient’s mother denied having observed faecal passage from the vagina.

A clinical diagnosis of H-type ARM was made and parents were counselled for admission where antibiotics were started for the child. The patient was scheduled for an examination under anaesthesia (EUA) and diversion colostomy the next day.

**Figure 2:**
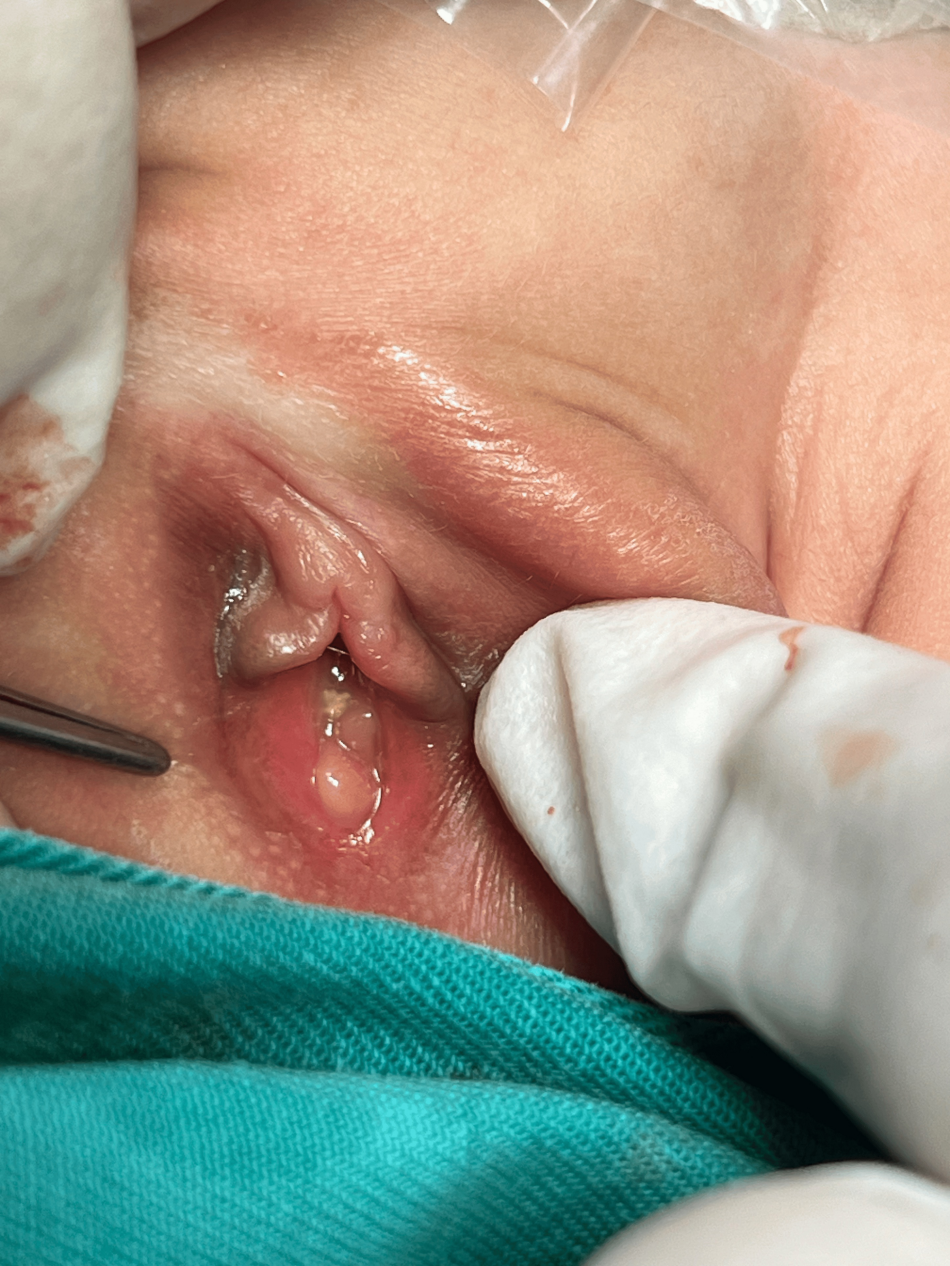
On examination under anaesthesia (EUA), faecal matter is observed emerging from the vestibule upon compression of the left labia.

Under anaesthesia, it was observed that the labial swelling and erythema had reduced compared to the previous day, correlating with more faecal matter passing at the posterior fourchette (Figure 2). The opening of the fistula could not be appreciated, thus attempts at probing failed. We were unable to visualise the fistulous opening from the anorectal examination which was otherwise unremarkable. A diverting left transverse loop colostomy was then fashioned. Surgery was uneventful and the child was nursed in the general wards post-operatively. Her post-operative recovery was smooth and she was discharged well with complete resolution of the labial swelling.

## Discussion

ARMs are one of the more frequent congenital anomalies encountered by paediatric surgeons occurring 1 in every 5000 live births [1]. It is often diagnosed at birth; however, it has an array of presentations. Currently, the Krickenbeck Classification System is used to classify these anomalies. The classification system helps healthcare providers to better communicate with each other, decide on management and predict the outcome [2]. Overall, ARMs are slightly more common in males compared to females with a ratio of 1.2 to 1 [1].

ARMs are thought to arise from abnormalities in embryonic development, with two main theories proposed. The first suggests that the urinary, reproductive, and gastrointestinal tracts initially share a common cloaca, and improper separation or migration of the anorectal septum around the seventh week of gestation leads to malformations. The second theory posits that abnormal migration of the rectum toward the perineum during development results in these defects. However, neither theory has been definitively confirmed by embryologic models, and further research is needed to clarify the precise mechanisms underlying ARMs [1].

H-type fistulas are categorised as rare/regional variants under the Krickenbeck Classification. Its incidence is reported to be 0.1-16% of all ARMs with a female-to-male ratio of 10:1. There is also a preponderance of cases within Asian countries particularly India, Japan, China, Kazakhstan, and Bangladesh [3]. The reason behind the preponderance towards the female population remains unclear [3]. Establishing a diagnosis at birth is rare, with a history of passage of stool from the vagina being the most common presentation in girls. In male patients, they may report passing urine from the rectum. Other reported symptoms include constipation and recurrent urinary tract infections. Although there have been reported cases of H-type fistula presenting as labial abscesses, it remains a rare clinical entity. It is unsurprising then that many patients may be mistreated for a labial abscess without having a proper diagnosis established.

Almost all reported cases of rectovestibular fistula in available literature present with left labial abscesses. In a retrospective study of five babies with similar conditions, Kim et al. reported only one patient who presented with a right labial abscess [4]. The pathophysiology behind the laterality of the abscess formation is not known.

Diagnosis can be made clinically paired with a suggestive history, confirmed with EUA and direct visual inspection with probing of the fistula tract. However, other diagnostic modalities include transperineal ultrasound, contrast study, dye injection into the rectum, vaginoscopy, proctoscopy and magnetic resonance imaging [5]. Barium study is found to be an acceptable first step diagnostic modality if the diagnosis is uncertain. Even if results are negative or equivocal, follow-up EUA should be made to look hard for a fistula especially if there has been a history of stool passage from the vagina [4].

We performed a diversion colostomy for our patient while letting the labial abscess heal. Further plans will be for a ligation of the fistulous tract. There are many reported surgical approaches to this condition. These include anterior sagittal anorectoplasty with or without colostomy, colostomy with interval fistula closure or single-stage fistulotomy and curettage [5]. Although reported outcomes are good in terms of anorectal control, the rate of recurrence has been reported to be 5-60% [3]. The final decision for the surgical approach should be based on the expertise of the paediatric surgeon.

For patients with rectovestibular fistula, a thorough evaluation for associated anomalies should be routinely performed. Associated anomalies including VACTERL-type anomalies can exist in 20-60% of cases and are much more likely in males; many females with H-type fistula have no other anomalies.

In comparison, a labial abscess without the presence of ARM is typically due to Bartholin’s gland abscesses, which are extremely rare in prepubertal girls, with only a few reported cases in infancy and neonates. They typically present as tender cystic swellings in the labial region and are often excluded from the differential diagnosis of labial masses in paediatric patients. Standard treatment involves incision and drainage, often followed by antibiotic therapy, with bacterial cultures sometimes returning negative due to prior antibiotic use. The prognosis is generally favourable, with most cases resolving without recurrence following appropriate management [6].

Although rare, this case highlights the importance of recognising the various clinical presentations of ARM. Unlike a conventional labial abscess, which typically arises from localised skin infections, bacterial overgrowth, or inadequate hygiene, this case was distinguished by the presence of faecal contamination within the vestibule. This unusual finding strongly indicated an underlying anorectal fistula, suggesting an abnormal communication between the rectum and external genital structures. The presence of stool in the vestibular region not only raised concerns about chronic contamination and recurrent infections but also highlighted the need for further diagnostic evaluation to confirm the presence and anatomical course of the fistula. This atypical presentation underscores the importance of considering ARMs in paediatric patients presenting with unexplained perineal abscesses, particularly in the absence of other common predisposing factors.

## Conclusions

H-type fistula is a rare subtype of ARM. There must be a high index of suspicion for this condition in infants who present with a labial abscess, and early referral to the paediatric surgeon is warranted. Red flags should be raised in patients who present similarly and those who deviate from the presentation of simple labial abscess which in itself is a rare occurrence in this age group.
